# Assessment of minor psychiatric morbidity, stressors, and barriers of seeking help among medical students at the University of Khartoum, Khartoum, Sudan

**DOI:** 10.11604/pamj.2020.35.87.17512

**Published:** 2020-03-24

**Authors:** Muwada Bashir Awad Bashir, Sara Omer Abdelazim Mohamed, Claude Ngwayu Nkfusai, Fala Bede, Olanrewaju Oladimeji, Joyce Mahlako Tsoka-Gwegweni, Samuel Nambile Cumber

**Affiliations:** 1 Discipline of Medicine and Surgery, Faculty of Medicine, University of Khartoum, Khartoum, Sudan; 2 Cameroon Baptist Convention Health Services (CBCHS), Yaounde, Cameroon; 3 Goergetown University's Center for Global Health Practice and Impact (CGHPI), TIDE-Cameroon Program, Bertoua, Cameroon; 4 Center for Community Healthcare, Research and Development, Abuja, Nigeria; 5 Department of Public Health, Walter Sisulu University, Eastern Cape, South Africa; 6 Faculty of Health Sciences, Durban University of Technology, Durban, South Africa; 7 Office of the Dean, Faculty of Health Sciences, University of the Free State, Bloemfontein, South Africa; 8 Centre for Health Systems Research and Development, University of the Free State, Bloemfontein, South Africa; 9 School of Health Systems and Public Health, Faculty of Health Sciences, University of Pretoria, Pretoria, South Africa

**Keywords:** Medical students, psychological morbidity, barriers, stressors

## Abstract

**Introduction:**

Medical education can be stressful and a source of psychiatric morbidity for medical students with the potential of causing serious professional and personal negative consequences. With the limited studies investigating this issue in Sudan, this study aimed at assessing psychiatric morbidity, determine stressors, evaluate mental health care seeking behavior and barriers to seeking help among medical students in Khartoum, Sudan.

**Methods:**

This was a cross-sectional study with data collection for a period of one month, during the survey. Following ethical clearance and administrative approval, 644 students who gave consent were selected randomly from the university of Khartoum’s faculty of medicine. The “12-General Health Questionnaire (GHQ12)” was used as a tool to assess prevalence of psychiatric morbidity, determine stressors and evaluate barriers to seeking mental health care among students for a period of a month.

**Results:**

The overall prevalence of psychiatric morbidity was 56% (n = 356). The mean score of the GHQ12 was 6.7. There was a statistically significant association between GHQ12-score and level of study (in medical school), age, student’s income (student financial allowance). Stressors mostly experienced by students were fear of academic failure, dissatisfaction with academic performance and examination stress. The most frequent barriers to seeking mental health care elicited by participants were fear of stigmatization 63% (n = 401), preference for dealing with the problem alone 60% (n = 379), fear of the unknown 59% (n = 365) and failure to recognize symptoms 58% (n = 366).

**Conclusion:**

Psychiatric morbidity is commonly experienced by students in medical school as can be seen from the high prevalence (56%). The reported high figures of psychiatric morbidity among medical school students points to the urgency for interventions to address this problem with potential for negative sequelae (personal and professional). Our findings suggest that interventions to improve the social and economic conditions of students in medical school as well as addressing stigma related to mental health and educating students to recognize signs and symptoms of psychiatric morbidity while making help accessible might go a long way to address this challenge.

## Introduction

Mental health disorders according to the World Health Organization (WHO) are one of the leading causes of disability worldwide. Mental health disorders rank three out of the ten leading causes of disability in people between the ages of 15 and 44 accounting for a growing burden of health inequality worldwide often demonstrated in different abnormal behaviors and communication patterns and defective perceptions and thoughts [1]. Evidence reveals that university students are subjected to more stressful conditions than general population. Medical training, in particular has been recognized as a stressful experience with medical students highly subjected to stressful conditions that exerts a negative effect on their academic performance, physical health and psychological wellbeing [2-4]. The demanding academic requirement of being a medical professional, the nature of the disciplines' tasks and environment that expose students to the suffering of patients and deaths, coupled with the workload and the required quality of the educational process are all risk factors that are found to create stress and negatively impact students' mental well-being [5, 6]. It has also been well proven that psychological stress could negatively impact students experience and level of professionalism both during medical training and future practice as medical officers engage in substance abuse behaviors or experience a recurrence of mental morbidities they developed earlier during their training and other manifestations of dishonest practice and patient's negligence [6]. Burnout which is a measure of psychological distress among doctors and medical practitioners is responsible for high figures of work quitting, absenteeism and low quality of life among practicing doctors and students, originating early during medical training and was found to be significantly associated with poor health outcomes and mental morbidities [6]. Despite the general expectation to have better access to mental health care, in fact medical students are less likely to receive proper mental care because of barriers of time availability, cost, inappropriateness of the available mental care, stigmatization and even students failure to recognize their own mental and psychiatric problems [7].

Causes of medical students' susceptibility to psychological stress varies from individual characteristics like personal coping mechanisms to general factors, some of which are social like financial obstacles, peer relations and support and absence from families while others are related to the effect of the educational process on students in addition to the disciplines' related barriers of utilization of mental health services by students such as the fear of documentation in academic record [6-9]. In Africa, studies of psychological distress and mental morbidities among students of medical studies have divulged worrisome figures that accounted for up to 86% with significant effects on students' medical performance [10-13]. In Sudan, the youth including tertiary students represents a major stratum in the society [14]. A study conducted in Khartoum revealed that half of medical students suffer from a degree of depression, anxiety or stress with significant implication for students' physical health and quality of the educational environment as determinant of the study outcome [15]. Studies conducted to address the exact causes, social and demographic implications of mental health problems and psychiatric morbidities among medical students in Sudan are few. In recognition of such a gap, this study aims at assessing minor psychiatric morbidity, experienced stressors, and barriers to seeking mental health care among medical students at the university of Khartoum, in the State of Khartoum, Sudan.

## Methods

This study was approved by the Research Ethics Committee of the Department of Community Medicine at the Faculty of Medicine, University of Khartoum where the study was conducted. The Faculty of Medicine, University of Khartoum has a total of 2343 students. Following administrative authorization, those who were willing to participate in the study from first academic year to the sixth academic year were invited to fill the questionnaire used for data collection. This was done for one month and it was anonymous and voluntary process without any form of coercion following consent. Participants could withdraw from the study at any time. Students were fully informed about the purpose and the nature of the study. The questionnaire was self-administrated with consenting students completing either the paper or electronic version of the questionnaire.

In consideration of the prevalence value of stress among medical students in a similar study conducted in Sudan, the prevalence value relied upon for the sample calculation was 50% [15]. Considering 95% level of precision, we came to a sample size of 586. Seeking for better sample representation and considering the lost data due to incomplete and improperly filled questionnaire we further increased the sample size by 10% arriving at final sample size of 644 students. To increase our study generalizability, systematic random sampling technique was adopted to include our study participants using the class list of registered students as our sampling frame properly completed questionnaires were numbered anonymously and processed by computerized systematic random sampling generator. The numbers of the questionnaires produced by the systematic random sampling generator were included for analysis.

The data collection tool used in this study was a voluntary self-administered questionnaire consisting of three sections. The first section captured socio-demographic data and included questions about personal and social characteristics. The second section was the 12 items General Health Questionnaire (GHQ12) which is a popular measure of psychological distress and minor mental morbidity developed by the British Scholar Goldberg in 1972. The third section was a questionnaire developed to assess stressors experienced by medical students and their perceived barriers to seeking help based on findings from literature review. The GHQ12 consists of 12 questions each of which has four answers scored from 1 to 4 [16]. Internal validity and appropriateness of the tool was done by pilot testing the questionnaire among 60 volunteer medical students prior to the commencement of the study and none of them was included in the study sample.

The GHQ-12 has high reliability and validity of testing for psychological distress and minor mental morbidities within the past few weeks with a 90% reliability based on the binary GHQ (0-0-1-1) scoring method that was used for the purpose of this study [17]. While the questionnaire has six reversed items and six non reversed ones, scoring gave the value of 1 to the most two symptomatic options and the value of zero to the least two symptomatic and the opposite was applied to the reversed items. The idea behind using this scoring method instead of the conventional Likert scoring system is to conceal the possibility of bias due to participant's tendency to choose either extreme or average values of the scale [16]. All items were added to a scale from 0 to 12 and the scale was summed to retrieve each participant's score. Aiming at overcoming the verity in the general health questionnaire threshold, the mean score of the participants of the study was used to determine the cut off point for morbidity status which was 6/7 in this study [18]. Accordingly, a score of 7 and above were considered positive for mental (psychiatric) morbidity.

Students' perception about the types of stressors they experience as medical students and barriers to seeking mental health services were assessed using a set of factors that have been identified from similar studies and literature review [19-21]. Students were asked to reflect on their perception of one of the listed factors as barrier and stress they are experiencing on a scale of: Strongly agree, Agree, Neutral, Disagree and Strongly disagree. Students who answered Strongly agree and Agree were counted as positive while Neutral, Disagree and Strongly disagree responses were considered negative. Participants were also asked to introduce non-mentioned factors that they perceive but not present in the questionnaire and to provide information about whether they seek any form of mental health services either medical or alternative care.

GHQ-12 scores, socio-demographic characteristics and students perceived barriers of access to mental health care and experienced stressors contributing to mental morbidities were descriptively presented as percentages among the sample of participants. Logistic regression method was used to test for association between participants' socio-demographic parameters and the outcome variable, presence or absence of mental (psychiatric) morbidity. In this method, the model gradually includes variables that are statistically significant in predicting the outcome variable of students' mental health status to be morbid or non-morbid. Data was analyzed using the predictive SPSS software version 24 for windows.

## Results

**General characteristics of study population:** six-hundred and forty-four (644) students participated in the study. Most of them were females 398 (62%) while males were 246 (38%). Students of the third academic year had the highest representation in the study sample 164 (26%), followed by students from the first academic year 133 (20%), second and fifth academic years had a relative equal representation of 103 (15%) and 100 (16%) and the least representation was from the sixth year 82 (12%) and the fourth 59 (9%) year students. The mean age of the participants was 20 (SD = 2.5) years old. Most of the participating students live with their families 477 (74%) while those who live alone in student apartments or with friends in flats were almost equal with a frequency of 72 (11%) and 75 (12%) respectively. Majority of the students, 258 (40%) belong to the income group of 500-1000 Sudanese pounds (SDG) per month, 228 (35%) to the income group of 1000-2000 SDG per month and the least 110 (17%) to the highest income group of more than 2000 per month (Table 1).

**Table 1:** Social characteristics and percentages of GHQ12-caseness

Socio-demographic Characteristic		Psychologically non-morbid (Psychiatric morbidity present) GHQ-12 Score ≤ 6		Psychologically morbid (Psychiatric morbidity Absent) GHQ-12 Score > 7	
		Count	%	Count	%
**Gender**	**Male**	131	36.8	115	40.2
	**Female**	225	63.2	171	59.8
**Age in years**		mean	20	mean	20
**Academic Level**	**First year**	70	19.7	63	22.2
	**Second year**	66	18.6	37	13.0
	**Third year**	96	27.0	68	23.9
	**Fourth hear**	25	7.0	34	12.0
	**Fifth year**	62	17.5	36	12.7
	**Sixth year**	36	10.1	46	16.2
**Monthly Income (Allowance)**	**Less than 500**	31	8.7	17	5.9
	**500-1000**	151	42.4	105	36.7
	**1000-2000**	108	30.3	120	42.0
	**More than 2000**	66	18.5	44	15.4
**Residence**	**With family**	272	76.4	203	71.0
	**With friends/flat**	37	10.4	38	13.3
	**Alone in dorms**	39	11.0	33	11.5
	**Others**	8	2.2	12	4.2

**Assessment of GHQ-12 score of participants:** the mean of the general health questionnaire score of the participants was 6.7 (SD = 2.3) considering a cutoff point of 6 for the mental (psychiatric) morbidity status, 356 (56%) of the students scored 7 or above and so demonstrated mental morbidity as opposed to 286 (45%) who scored 6 and below. To decide which variables is majorly predicting student's mental health status using logistic regression method, we used different cut off points to test for variables' inclusion in the models. The variable that was included in models from all cut off points that have a major influence in the student mental health status was the academic year. Factors that significantly affect students' mental health status outcome according to the model with the highest predictive capacity were academic years of the student (p-value = 0.002), age (p-value = 0.01) and students' monthly (allowance) (p-value = 0.03). Gender (p-value = 0.83) and residence (p-value = 0.54) were not significantly associated with student mental health status (psychiatric morbidity). The odds of psychiatric morbidity was lower with increase in age (β = -0.229, Odd Ratio = 0.733, p = 0.01) implying younger students were more likely to present with mental health problems. Taking first year students as reference, the odds of psychiatric morbidity for sixth year students was more than twice relative to first year students (β = 0.953, Exp β or Odd Ratio of 2.6). There was an observed trend; with the odds of psychiatric morbidity which increased with increase in academic level. Students with monthly income <500 Sudanese pounds were more likely to present with psychological morbidity relative to those with monthly income (allowance) above 2000 Sudanese pounds (β=0.547, Exp β or Odd Ratio of 1.7, p = 0.03) (Table 2).

**Table 2:** Factors associated with medical students’ mental health outcome

Variables in the Equation		β	S.E.	Exp (β)	95% C.I. for Exp (β)	Wald test p-Value
**Monthly Income (Allowance) in pounds (sudanese)**						0.03*
	**More than 2000**	REF	REF	REF	REF	
	**1000-2000**	-0.34	0.342	0.711	0.364 - 1.392	
	**500-1000**	-0.168	0.196	0.846	0.575 - 1.243	
	**Less than 500**	0.547	0.255	1.728	1.049 - 2.847	
**Age in years**		-0.229	0.082	0.743	1.070 - 1.477	0.01*
**Academic Level**						< 0.01*
	**First year**	REF	REF	REF	REF	
	**Sixth year**	0.953	0.365	2.593	1.267 - 5.308	
	**Fifth year**	0.332	0.362	1.393	0.686 - 2.832	
	**Fourth hear**	0.107	0.269	1.113	0.657 - 1.885	
	**Third year**	0.461	0.398	0.886	0.625 - 1.147	
	**Second year**	-0.653	0.328	0.521	0.274 - 0.990	

1 USD = 55 sudanese pound

* statistically significant at p < 0.05

**Figure 1:**
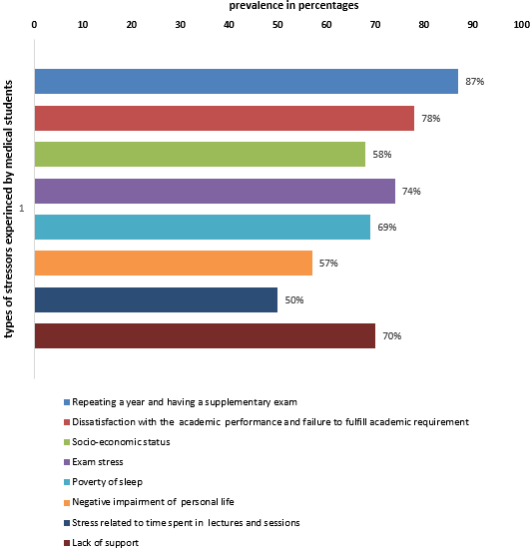
Stressors experienced by students in medical school

**Assessment of stressors experienced by medical students:** repeating a year and having a supplementary examination 327 (87%) and dissatisfaction with the academic performance and failure to fulfill academic requirement 310 (78%) were the most frequent stressors given by medical students. Other stressors like poor income (allowance) 262 (68%), examination stress 251 (74%) and lack of sleep 260 (69%) were also highly reported by students as stressors perceived to risk their psychological health during the period of medical training. More than half, 367 (57%), of the students mentioned that medical school is negatively impairing their personal life while 322 (50%) revealed their psychological distress by the time spent in lectures and sessions. Lack of support was a stressor reported to be experienced by 456 (70%) of the medical students (Figure 1).

**Figure 2:**
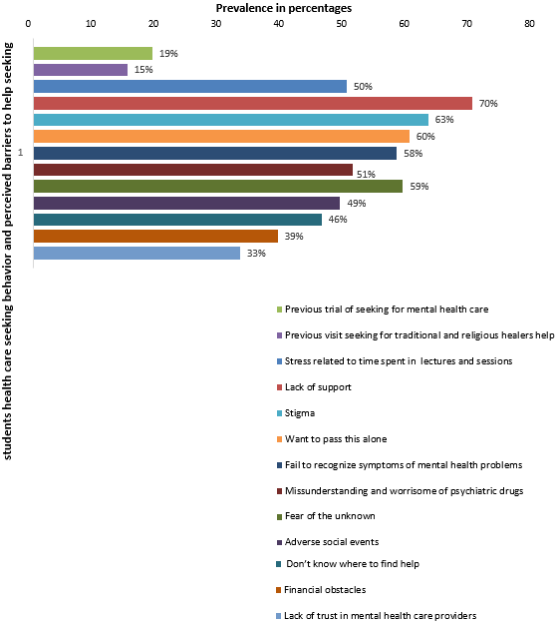
Medical student's heath care seeking behavior and perceived barriers to mental health care

**Medical student mental health seeking behavior and barriers:** few, 123 (19%), reported a willingness to seeking mental help as oppose to 454 (71%) who did not. Fewer, 98 (15%), mentioned actually seeking any form of help from traditional or religious healers. With regards to barriers evoked by participants preventing them from seeking help for mental health problems, 401 (63%) of the medical students mentioned fear of stigma, 379 (60%) of the students stated a preference to deal with problems alone when they do not feel psychologically well and 366 (58%) mentioned that they failed seeking help because they do not recognize or know what signs or symptoms may be related to psychological problems. In addition, fear of potential effects of medicines used for management of psychiatric conditions was reported by 326 (51%) of the student as barrier, more than half, 365 (59%), stated being worried of uncertainties surrounding mental health diagnosis i.e. fear of the unknown and 313 (49%) of the students said that adverse social events might be the reason for not seeking mental health care. Not knowing where to seek help for psychological problems was a reason mentioned by 294 (46%) students for failing to seek care for mental health problems and financial constraints was a reason elicited by 249 (39%) as a potential barrier to seeking mental health care. Lack of trust in health system (mental health care providers) was the barrier stated by 211 (33%) meanwhile 266 (42%) of the students attributed failure to seek help to traditional and religious beliefs (Figure 2).

## Discussion

This study aims at assessing minor psychological morbidities and its association with socio-demographic characteristics of medical students in Sudan. It also explored medical students' mental health care seeking behaviors and perceived barriers and the different types of stressors perceived by them using the general health questionnaire as a reliable tool of assessment that was formerly used by the World Health Organization as a screening tool [22]. The study found that more than half of the students are psychologically distressed (56%). The findings are close though slightly higher than the findings from another study conducted among medical students in Sudan that revealed a 50% prevalence and another study in Iran that reported similar percentage of psychological morbidity among medical students [8, 15]. However, this study has completely matched results from a study conducted in Turkey [23]. Generally, the percentages of psychological distress among medical students in Sudan is higher than the figure reported from other studies conducted in the developed world [6, 24]. When also compared to studies conducted in Asian countries of similar socio-economic set up, the figures reported in our study were higher than similar studies done in Nepal, Bangladesh and India [25-27]. However, in studies from Saudi Arabia and Egypt our study findings were lower in prevalence of psychological distress and morbidities among medical students [28-30].

The study showed that student's probability to develop psychological morbidities increases as they progressed in academic years resulting in most senior students having the highest chance to develop negative psychological outcomes. Although such findings were not tested by the objectives of this study, possible explanation for it is that, medical education in the university of Khartoum is structured in variant patterns along the college time. The assessment systems, subjects and academic load in each year vastly differ as a requirement from a year to the next. This fact may conceal students coping mechanism over time making them not to benefit from the feedback on the effectiveness of their learning methods and exposing them to a renewable source of stressors in form of fear from the unexpected in the absence of the proper guidance and academic support. This finding is in line with similarly reported worsening mental health outcomes in medical schools in studies from United Kingdom, Turkey and Norway [31-33]. Other studies have reported quite different conclusions when it comes to academic level and its impact on students' mental health outcomes [8, 34]. It was found that advanced levels of academic study in medical schools is associated with lower prevalence of psychological morbidities; a finding that is in contrast with the findings of our study.

The finding that younger age and higher income level are significantly protective factors against negative psychological outcomes among medical school reported in this study are consistent with figures reported from studies conducted in Egypt and Saudi Arabia [28]. The findings relevant to expenditure might mirror the fact that, financial obstacles being key stressors for medical students as a result of the demanding nature of the medical school requirement considering the low socio-economic status of Sudan. Regarding the psychological stressors experienced by medical students in this study, the finding that the most frequently reported stressor is fear of repeating an academic year or having a supplementary exam, failure and dissatisfaction with the academic performance and exam stress are alike to findings reported in many other studies. Examinations which are a necessary tool for assessment in most medical schools in Sudan are perceived in different ways by students as some of them consider it as a stressor while others benefit from it as a learning method [26]. However, circumstances related to the setting and timing of examinations, lack of considerations and support for students with medical, psychological and learning disabilities in assessment methods and the extent of stringency of laws and regulation related to students' academic assessment are differential factors in medical students' perception of its academic related stress.

Psychosocial stressor could be related to family and community expectations, lack of entertainment, future concerns and lack of sufficient social support from family and friends due to distant learning conditions or time availability issues during medical education. Other causes might be related to financial difficulties experienced due to the demanding needs of medical education in Sudan in the face of the low-income status of the majority [26]. In this view, the reported high levels of lack of social support, personal life impairment and economic stresses in this study, mirrors an extensive need for medical school environment enhancement to be more psychosocially friendly by adding recreational and social assistance services. Universities should structure programs into reasonable curricula and academic load that students have spare time for themselves personally and physically. Social counselors and educational methods and structures that are more exciting and interesting for students in addition to the previously mentioned measures, was proven to significantly reduce negative mental health and psychological outcomes among medical students [35].

From a general prospect, these figures are justifiable by the recent paradigm shift witnessed by the medical education field in Sudan. After 1990 and the introduction of the new admission policy to higher educational institutions in Sudan including medical schools was adopted, students were able to get admission into medical schools based on their social and political affiliations and private admissions was approved to support medical schools financially. This fact resulted in crammed classes in medical schools where students are of extreme difference in terms of their intellectual capacities and key skills like language and a few competing in a passive educational process with lack of sufficient qualified trainers and educators. Educational environments including hospitals where students are clinically trained are lacking the basic assistance and set up for educational process. In addition, students are further faced with a future of difficult employment opportunities aggravated by the low socio-economic status of the country where they must deal with their patients' difficult socio-economic status as care givers who tackles their medical, social and financial hardships [36, 37].

Perceived barriers to mental health care among students in this study mirrors a major existence of public stigmatization even among supposedly highly knowledgeable social group about mental health like medical students. The effect of this on future career was not explicitly tested in this study. However, this presents a fertile field of research to study the buildup of mental health stigmatization and its projection on health seeking behavior facilitation and hindrance given the unique knowledge variation among this group. Other highly frequent barriers were related to the fear of stress from health seeking act itself, where students prefer to rely on themselves in dealing with their psychological problems and in the ability to recognize their need for help and over estimation of self-coping mechanisms. The least present factor was accessibility issues whether financial or physical, concerning provider's characteristics and lack of knowledge about mental health issues. This reveals a significant thematic variety in the key barriers to mental health care among medical students in Sudan. Although barriers related to fear of being a burden to others and ability to express emotions were not explicitly revealed by the students, this may be explained by the strong family and social ties characterizing the Sudanese community or the absence of questions that reflect such themes of barriers in the study questionnaire explicitly.

This characterization of the barriers perceived by medical students in this study is comparable to the findings of a review conducted among young population for the same outcome [38, 39]. However, this study did not approach mental health facilitation factors which is an arena that needs to be further enriched with future research. This study is limited by the fact that it did not take into account the variety in the faculty teaching approach in different levels which may affect the outcome of interest and also the fact that the data collection method in this study was a self-administered questionnaire which allows for subjective bias introduction into the findings. Even though the cross-sectional design of this study might limit its causal inferential findings, the stepwise modeling analysis method and the use of a standardized method of assessment gives it a unique value of credibility as an additive effort to the scarce arena of mental health research among the young population generally and medical personnel in Sudan. This study may be considered as a precursor for further medical education-oriented quality and population wide prospective researches in Sudan to further investigate medical students' challenges and facilitations towards better wellbeing and quality education.

## Conclusion

High figures of psychological morbidity are being present among medical students in Sudan. The factor that is the most predictor for student’s psychological outcome was the academic year. Students in the early academic years of medical schools are more likely to acquire negative mental health outcomes than students in advanced years although monthly income and age have significant effect on students' mental health status. Medical students are experiencing wide variety of stressors in medical school and the most burdening are those related to academic performance requirements and outcomes. Low figures of students' seeking mental health care before with high reported figures of barriers perceived by students which are mostly related to issues of public stigmatization, tendency to rely on self for handling such problems or accessibility as well as provider's knowledge and trust barriers. The high figures of psychological morbidity, experienced stressors and barriers to mental help seeking experienced by medical students, revealed in this study are indicative for an urgent need for mental health-oriented interventions to be considered in medical schools. Our findings suggest that interventions to improve the social and economic conditions of students in medical school as well as addressing stigma related to mental health and educating students to recognize signs and symptoms of psychiatric morbidity while making help accessible might go a long way to address this challenge.

### What is known about this topic

- Depressed medical students' use of mental health services and barriers to use; the reliability of the twelve-item general health questionnaire (GHQ-12) under realistic assumptions;
- Psychiatric morbidity among third year medical students at the Ain Shams University;
- Perceived barriers and facilitators to mental health help-seeking in young people: a systematic review.

### What this study adds

- The factor that is the most predictor for student psychological outcome was the academic year; students in the early academic years of medical schools are more likely to acquire negative mental health outcomes than students in advanced years although monthly income and age have significant effect on students’ mental health status;
- Low figures of students’ seeking mental health care before with high reported figures of barriers perceived by students which are mostly related to issues of public stigmatization, tendency to rely on self for handling such problems or accessibility as well as provider’s knowledge and trust barriers;
- The high figures of psychological morbidity, experienced stressors and barriers to mental help seeking experienced by medical students, revealed in this study are indicative for an urgent need for mental health-oriented interventions to be considered in medical schools.

## Competing interests

The authors declare no competing interests.
